# Stercoral Colitis Secondary to Opiate-Induced Constipation

**DOI:** 10.7759/cureus.50511

**Published:** 2023-12-14

**Authors:** Pranav R Chepyala, Anjali R Daniel, Murdoc B Gould, Muneet Gill, Latha Ganti

**Affiliations:** 1 Biomedical Sciences, University of Central Florida, Orlando, USA; 2 Biology, Emory University, Atlanta, USA; 3 Chemistry, Rollins College, Winter Park, USA; 4 Emergency Medicine, Brown University, Providence, USA; 5 Medical Sciences, The Warren Alpert Medical School of Brown University, Providence, USA; 6 Emergency Medicine and Neurology, University of Central Florida College of Medicine, Orlando, USA

**Keywords:** opiate-induced constipation, constipation, chronic constipation, stercoral ulcer perforation, stercoral colitis

## Abstract

The authors present the case of a 62-year-old woman who had stercoral colitis secondary to opiate use for rheumatoid arthritis leading to chronic constipation. Computed tomography imaging demonstrated stool along a significant length of the colon. Stercoral colitis is a seldom suspected cause of severe abdominal pain. Although constipation may seem benign, when it gets to the level of a stercoral colitis, mortality due to colonic perforation is a very real concern. The authors review the presentation, risk factors, and management of stercoral colitis.

## Introduction

Stercoral colitis is characterized by inflammation and ulceration of the colonic wall due to fecal impaction [1]. Patients with stercoral colitis often present with abdominal pain, distension, constipation, and sometimes rectal bleeding. These symptoms are nonspecific and can mimic other colonic diseases, necessitating a high index of suspicion for diagnosis. The pathogenesis involves increased intraluminal pressure due to fecal impaction, leading to ischemia and ulceration of the colonic wall. Stercoral perforation occurs when pressure necrosis results from the fecaloma [2].

The sigmoid colon is most commonly affected due to its narrow lumen and vulnerability to pressure-induced ischemia. Microscopic changes are observed in stercoral colitis, including transmural inflammation and necrosis. Risk factors for stercoral colitis include any condition that cause fecal impaction, including chronic constipation, neurogenic bowel, psychiatric illnesses, and opiate use. Additionally, advanced age, immobility, and dehydration are also risk factors [3].

Treatment focuses on relieving fecal impaction and managing colonic inflammation. A combination of disimpaction, laxatives, enemas, bowel rest, intravenous hydration, and electrolyte correction is typical for non-perforated cases. Surgical intervention is considered in cases of perforation or failure of conservative management.

Complications include colonic perforation, sepsis, and shock. Delayed treatment significantly increases the risk of complications, including fecal peritonitis and abscess formation. The prognosis depends on early recognition and treatment. Timely management generally results in favorable outcomes, but delayed treatment can lead to high mortality, especially in cases with complications.

## Case presentation

A 62-year-old female with a past medical history of rheumatoid arthritis, hemorrhoids, and chronic constipation secondary to opiate pain medication use presented to the emergency department (ED) for constipation. She stated her last bowel movement was three days prior; however, her stool was hard, and she felt like she was incompletely evacuating. She explained while she had the urge to go, she was unable to have a bowel movement. She reported taking oxycodone daily for pain secondary to her rheumatoid arthritis. Her other medications were methotrexate and loratadine. She also reported taking bisacodyl laxative as needed, and her last dose was three days ago. The patient endorsed associated lumbar back pain and nausea; however, she denied fevers, chills, chest pain, shortness of breath, vomiting, abdominal pain, rectal pain, blood in stool, or dysuria.

Her vital signs were as follows: blood pressure 171/94 mmHg, pulse 99 beats per minute, pulse oximetry 99% on room air, temperature 97.9°F, and respirations 18 breaths per minute. Her laboratory tests are summarized in Table 1 and were essentially unremarkable. Urinalysis revealed normal result. 

**Table 1:** Patient's laboratory values

Laboratory test	Value	Reference range
Sodium	136	136-145 mmol/L
Potassium	3.5 L	3.7-5.1 mmol/L
Chloride	105	98-107 mmol/L
Carbon dioxide	25	21-32 mmol/L
Anion gap	9.5	Less than 14
Blood urea nitrogen	8	7-18 mg/dL
Creatinine	0.56	0.55-1.3 mg/dL
Glucose	117	74-106 mg/dL
Calcium	8.9	8.4-10.1 mg/dL
Total bilirubin	0.3	0.2-1.5 mg/dL
Aspartate aminotransferase	16	10-37 unit/L
Alanine aminotransferase	15	12-78 unit/L
Total alkaline phosphatase	97	45-117 unit/L
Total protein	8.2	6.4-8.2 g/dL
Albumin	2.9	3.4-5.0 g/dL
Lipase	48	20-140 unit/L
White blood cell count	7.7	4.0-10.5 10^3^/u
Hemoglobin	11.5	11.2-15.7 g/dL
Hematocrit	35.1	34.1-44.9%
Platelet count	395	150-400 10^3^/uL

**Figure 1:**
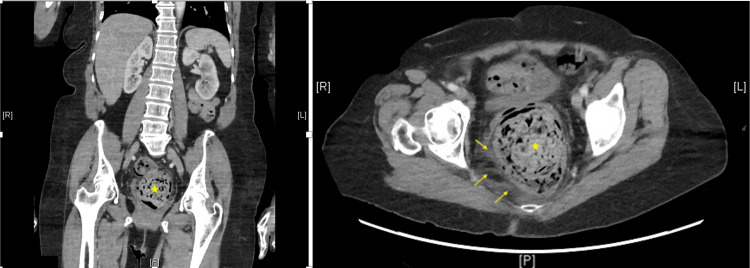
Coronal and axial views of CT scan with contrast of the abdomen and pelvis reveals large rectal stool burden (star) with mural thickening and perirectal inflammatory changes (arrows) CT: computed tomography

Computed tomography (CT) scan with contrast of the abdomen and pelvis revealed large rectal stool burden with mural thickening and perirectal inflammatory changes, consistent with stercoral colitis (Figure 1). Also noted is air in the rectal wall (pneumatosis).

Due to the extent of the obstruction, the patient was admitted to the hospital. She was initially made nil per os (NPO), hydrated with intravenous saline. The patient also received an enema and underwent manual disimpaction, followed by polyethylene glycol administration to assist with fecal evacuation. The patient did have bowel movements after this regimen. The patient was monitored for signs of sepsis, which she did not develop. A pain management consult was obtained to wean the patient off of daily opiates. The patient was discharged on hospital day 4, without any complications.

## Discussion

A retrospective review of 49 patients noted that the rectosigmoid colon was the most frequently involved segment in stercoral colitis. CT findings typical of stercoral colitis include dilatation >6 cm, wall thickening >3 mm of the affected colon segment, pericolonic fat stranding, mucosal discontinuity, and presence of free air, free fluid, and pericolonic abscess. The sign most related with mortality was the length of the affected colon segment >40 cm [4,5].

A cohort study of 452 mild and 93 moderate-severe cases confirms that the sigmoid is the most frequently involved segment. Factors associated with perforation included slightly increased wall thickness (6.4 vs. 5.7 mm, p=0.03), opiate use (50 vs. 23%, p=0.04), and disease-specific mortality (11 vs. 0%, p=0.04) [6]. Mortality in this cohort was 11%.

Management of stercoral colitis includes disimpaction (either manual or endoscopic), laxatives, and enemas. Mortality from stercoral colitis has been reported to be between 17% and 30% [6-10], and this is driven by cases with perforation, which is the single biggest risk factor for mortality. Once perforation occurs, surgical intervention is necessary to resect the segment of the colon involved. Most often, this involves resection of the sigmoid colon followed by an ileostomy.

Stercoral colitis is actually not an uncommon ED presentation. A recent retrospective cohort of 269 patients spanning three years from a regional health system found that while the most common chief complaint was abdominal pain (present in more than one third of the cohort), abdominal pain was actually documented as absent in two thirds of cases [10]. In these cases, if a CT were not done (due to lack of abdominal pain), the diagnosis of stercoral colitis would have been missed. Interestingly, 10% of patients discharged home returned to the ED within 72 hours. The authors note that in general, patients were not given specific instructions on how to manage the root cause, their constipation. This is an important point, as we will likely see more stercoral colitis given the increasing numbers of at-risk populations including the elderly, the infirm, and those on opiates.

## Conclusions

Stercoral colitis is a potentially fatal condition that requires prompt recognition and management. Opiate use is a significant risk factor that has become more prevalent. Early intervention is crucial to improve patient outcomes and reduce the risk of life-threatening complications.
